# Present-Day Mass Tourism: its Imaginaries and Nightmare Scenarios

**DOI:** 10.1007/s12115-020-00499-y

**Published:** 2020-07-19

**Authors:** Rob Kroes

**Affiliations:** Utrecht, Amsterdam The Netherlands

**Keywords:** Commodification paradox, Time travel, Sites of nostalgia, Lieux de mémoire, Global tourism, Disney-fication, Civil-rights tourism, Environmentalism, Utopian/dystopian imaginaries

## Abstract

Present-day mass tourism uncannily resembles an auto-immune disease. Yet, self-destructive as it may be, it is also self-regenerating, changing its appearance and purpose. They are two modes that stand in contrast to each other. We can see them as opposites that delimit a conceptual dimension ordering varieties of present-day mass tourism. The first pole calls forth tourism as a force leaving ruin and destruction in its wake or at best a sense of nostalgia for what has been lost, the other sees tourism as a force endlessly resuscitating and re-inventing itself. This paper article highlights both sides of the story. These times of the Covid-19 pandemic, with large swathes of public life emptied by social lock-down, remind us of a second, cross-cutting conceptual dimension, ranging from public space brimming with human life to its post-apocalyptic opposite eerily empty and silent. The final part of my argument will touch on imagined evocations of precisely such dystopian landscapes.

This is what present-day tourism has brought us. As Oliver Hardy would have it, in one of the many films starring him and Stan Laurel: Another nice mess you got us into. Photographs amply illustrate this. They show us the congestion, even back-ups, en route to the top of Mount Everest. Or the dense forest of outstretched arms and selfie sticks that prevent us from seeing eye to eye with Da Vinci’s Mona Lisa. They show all these cruise ships, ready to sail from Venice, New Orleans and other such ports-of-call, holding out the promise of fulfillment of our innermost private dreams and longings. Among them the classic dream that inspired travel in the days of the “grand tour,” seen as part of the education of aristocrats, of either noble or moneyed background, the elite of the happy few of their time. In today’s mass tourist version, all such dreams have been subverted and turned into their nightmarish opposite. Hordes of tourists now swoop down on places never meant to cope with their numbers. It may remind us of Henry James’s sense of horror when confronted with the mass of immigrants setting foot on Ellis Island. In *The American Scene,* written following a return visit to his native country and presenting a view of America seen through the eyes of the quasi-European that James had become, he compared the influx of immigrants to a “visible act of ingurgitation on the part of our body politic and social.” He goes on to ponder “the degree to which it is his American fate to share the sanctity of his American consciousness, the intimacy of his American patriotism, with the inconceivable alien … an apparition, a ghost, … in his supposedly safe old house.”^1^ This is an apt description of what inhabitants of today’s tourist destinations, ago-old port cities like Venice or Amsterdam, must be feeling in the face of the hordes of visitors dumped by one cruise ship following another. Admittedly, the visitors today are tourists, not immigrants, but they must strike a Jamesian sensibility in similar ways. And according to alarmed newspaper reports local resistance and protest in tourism’s most favored places is growing apace. The current buzzword in city government circles is over-tourism. As a piece in *Atlantic* magazine on “the Dutch war on tourists” put it: “The Dutch have suffered some brutal occupations, from the Roman empire and Viking raids to Spanish and Nazi rule. But now they face an even larger army of invaders: tourists.”^2^ In the era of cheap flights and Airbnb, their numbers are staggering. Some 19 million tourists visited the Netherlands last year, more people than live there. For a country half the size of South Carolina, with one of the world’s highest population densities, that is a lot. The problem for Amsterdam, in its starkest form, is a matter of survival as a working, residential city, rather than as a playground for tourists to trample underfoot. Venice may be closer to meeting that fate, with its residential population dwindling. And so may New Orleans after Katrina. In *New Orleans: An American Pompeii?*, Lawrence N. Powell demonstrates that the rebuilding of New Orleans’ infrastructure, which had been long due for an extreme makeover, is in danger of crossing over the fine line separating opportunity from opportunism, whether it be the opportunism of commercialism or racism or a combination of the two. The recovery of New Orleans, as Powell argues, may have resulted in one of those ‘lost cities’, like Pompei, that have been restored solely as sites of tourism and myth.^3^

Present-day mass tourism uncannily resembles an auto-immune disease. In a fevered feeding frenzy, it turns in upon itself eating away at the very tissue meant to be preserved. Like locusts swarming, tourism is seasonal, swooping down, leaving devastation in its wake. Yet, self-destructive as it may be, it is also self-regenerating, changing its appearance and purpose. The destruction side of the story I will tell here relates to an iron law in economics, the commodification paradox: there are things that by general consent are deemed of high intrinsic value yet are inconsistent with the economic logic of price and exchange value, or for that matter, the conceptual universe of economic goods and commodities. Once exposed to that logic, they vanish like snow melting in the sun. By way of examples, we need only think of exquisite geographic spots or authentic historic settings and see them vanish forever when opened to consumption by the many. The other half of my story offers redemption, in a post-modern vein. It explores the imitative behavior of tourists seeking reiterations of pleasures they have seen or heard described, if not vicariously experienced through mass advertising. Here the main vector of tourist behavior is not the quest for the pristine and virginal, but rather the urge to do as others did, and to join the multitudes who went before, all engaging in such acts of quasi-individuation as taking a selfie as proof of one’s presence. The ultimate self-ironizing version – post-modern before its time - is the classic graffiti telling us that “Kilroy was here”. Well, he wasn’t; yet clearly someone was wishing to leave proof of presence, tongue-in-cheek, in an implied wink to the many Kilroys yet to come. Clearly these two modes of tourist pleasure stand in contrast to each other. We can see them as opposites that delimit a conceptual dimension ordering varieties of present-day mass tourism. One polar end we may call – in an echo of a 1910s’ cliffhanger film series, *The Perils of Pauline* – “the perils of pristine.” The other end we shall call “the pleasures of post-modernity.” The first pole, then, calls forth tourism as a force leaving ruin and destruction in its wake or at best a sense of nostalgia for what has been lost, the other sees tourism as a force endlessly resuscitating and re-inventing itself.

## The Perils of Pristine

There may be no better way to illustrate the tragic paradox inherent in the human enjoyment of the pristine than the case of George Bird Grinnell, prominent early American conservationist. He had made it out West just in time to watch one era fade into another, on the eve of the Transcontinental Railroad opening the West to the many interests that had been eagerly eying it, and before Buffalo Bill’s mastery at turning contemporary history into the stuff of spectacle and mass entertainment had begun to re-write the epic of the West. Grinnell was among those who had an early awareness of the need, if not the moral duty, to preserve natural habitats and wildlife. Perhaps his greatest legacy is Glacier National Park in Montana, which he did more than anyone else to help protect, and where Mt. Grinnell now looks down on Grinnell Glacier. By the time of his last trips, in the 1920s, it was no longer the wild place he had first encountered. As he wrote to the soon-to-be-famous young conservationist Aldo Leopold, “While I have never regretted what I did in this matter because of the pleasure those parks give to a vast multitude of people, still the territory that I used to love and travel through is now ruined for my purposes.”^4^ This tragic awareness that the democratic sharing of his pleasures inescapably ruined them “for his purposes” lies at the heart of the conundrum that I flippantly call the perils of pristine.

Yet, undeniably, one perennial force moving people temporarily to leave home and hearth and go out into the wider world is the urge to explore and discover, to go where no-one has gone before, in hopes of striking upon the terrestrial paradise. And more than that, upon their return home, to engage in the games of one-upmanship that we all play, bragging about that pristine little beach or that bucolic little restaurant off the tourist track. We thus feed the mass reservoir of tourist longing, keeping people leafing through the pages of travel magazines, with one tantalizing view after another of places untouched by tourism. As one such magazine promises: We create memories. Rather a sophisticated view, in fact, coming as it does from a travel agency. For indeed: rather than promising novel experiences and new discoveries, the slogan anticipates the next stage: the translation of travel experiences into memories. Memories which we then share with others, leading them to follow in our footsteps – finding that pristine beach, that recondite little restaurant. Rather than *creating* memories it has become a matter of *re-creation*, in whichever sense of that word. Those following in our footsteps re-create *our* memories while making them *their* own. This is how tourism translates into a mass phenomenon, turning private memories into commodities advertised and held up for imitation. Peddling what are basically second-hand goods tourism endlessly recycles the standard tourist fare, boosting tourist numbers while ruining what made the tourist herds flock together in the first place. We may call the driving force here, in a Freudian vein, memory envy. The travel agenda in such cases is essentially of this type: Been there, Seen it, Done that. It has become a matter of ticking off places to go, to see, and produce a selfie. Today’s travel destinations are literally “lieux de mémoire”, to be visited before they are remembered, placed on the map by other people’s memories.

Things are different in the case of nostalgia. If memory still plays a role, it is the remembrance of things past, or more crucially of things irretrievably lost, yet awaiting their re-imagining. The setting is Proustian rather than Freudian. If it calls for travel, it is time travel, temporal and imaginary, rather than geographical. It can be done as an act of individual imagination, without ever leaving one’s armchair. One can, for instance, sit listening to Aaron Copland’s music, such as his *Rodeo*, while before one’s mind’s eye a film screen is widening to vast panoramas of the American West. If this is travel, it is a matter of the power of our imagination. Yet, in today’s world, our every wish can be accommodated by the market, and time travel now fills pages of tourist guides. If with the advent of the U.S. Interstate system, from the late 1950s on, fabled stretches of highway like Route 66 lost their *raison d’être,* and were left for weeds to take over, stretches are now – *nostalgically* – put back into use. Tourists from the US and Europe, their heads filled with Western imagery, from wide-screen cinema, songs from musicals, photographs, are now catered for with organized tours. They will find their Harley Davidsons waiting and off they go, in search of an America gone forever, yet now to be nostalgically revived. Route 66 has been re-invented as our collective present-day memory lane.

Examples abound of this happening. Civil-War battle fields, with historical re-enactments thrown in to the hearts’ content of visiting history buffs, the Appalachian Trail, freshly done up, with the added bonus of Bill Bryson’s dry wit reporting on his revisit of the trail, Crevecoeur’s travels in America as revisited in Jonathan Raban’s *Hunting Mister Heartbreak*, immersions in the history of railroad hotels like the Posada Hotel in Winslow Arizona (while hearing in one’s head the lyrics of “Standing on a corner in Winslow Arizona,” sung by the Eagles), or a stay at mountain resorts such as Bretton Woods, feeling the spectral presence of great minds like Lord Keynes’s putting together the pieces of a new world order in the mid-1940s.^5^ Such imaginary time travel is probably what the great Dutch historian Johan Huizinga had in mind when he tried to find words for the historic epiphany he experienced when entering Cologne cathedral from the bustling city center, in pre-World-War II days. To Huizinga it felt like stepping back into the peace and quiet of the Middle Ages. Time seemed to fall away. He described it as the quintessence of the *experience* of history, as an act almost of world renunciation in exchange for the order of the monastic, medieval world.

A contemporary version of this quest for sites of nostalgia is the Spa, the Grand Hotel, the fabled watering holes across the map of Europe, playground and meeting place for the international upper crust. They are sites of nostalgia today, remnants of a past long gone, vanished along with the “Belle époque” whose outward face they represented. Yet at the same time, vanished they may be, they refuse to be laid to rest. A cultural revival is afoot, in books and films, opening the doors of the past to our nostalgic promptings. The prime exhibit may well be a cinematic masterpiece, *Russian Ark*, from 2002. In one uninterrupted, long take – a technical tour de force – the director, Alexander Sokurow, takes us through three centuries of Russian history, set in the labyrinthine maze of the Hermitage in St. Petersburg. If this already is a trip that no travel agency can rival, the concluding moments give form and face to the process of history turning into instant nostalgia. While a magnificent ball is coming to an end and the many guests start flowing down the stairways, the camera wanders off to a door opening on the river Newa, with mist rolling in. It is an ominous closing image of an era coming to and end, engulfed by forces of darkness. It appears as if nostalgia for an era closing forever is shown here at the point of its formation.

In this general vein of nostalgic revisits to memory sites, a few more cases bear mentioning. The Grand Hotel as an emblem of a European cultural era has been stunningly brought back to life in Wes Anderson’s *Grand Budapest Hotel* (2014), with all the sense of decorum, punctilious rituals, and deference to status differentials. It is the world that that quintessential central-European author Stefan Zweig conjured up in *Die Welt von gestern* (*The World of Yesterday*), the book he wrote in self-chosen exile in Brasil in 1942.^6^ He committed suicide there, while his beloved Europe succumbed to Nazi totalitarianism. Wes Anderson dedicated his film to the memory of Zweig. More than that, he has Zweig make a fictional appearance in the closing scenes of the film, where he is shown reminiscing with the hotel’s current owner about his predecessor, Monsieur Gustave, the man who had truly embodied the world of the Grand Budapest Hotel. Although, as his successor clarifies, it had not even been Monsieur Gustave’s world: “His world? No, his world had vanished before he ever entered it. He certainly sustained the illusion with marvelous grace.” So, in this game of ever receding illusions, the true spirit of nostalgia is beautifully captured, and shown as the mirage it is.

Using a different medium, the novel rather than film, Dutch novelist Ilja Leonard Pfeijffer added to the metaphoric power of the Grand Hotel as standing for a certain idea of Europe when he called his latest novel *Grand Hotel Europe* (2019).^7^ Rather than an attempt at revival, though, showing a European cultural era at full swing, it catches it as it fades away, as in a yellowing old photograph. The hotel guests are like assorted relics from a bygone era. The novel’s protagonist seeks refuge among them to lick his wounds after the break-up of a relationship with Clio, an Italian art historian named after the Greek muse of history. The sense of things coming to an end, winding down, pervades the novel. More than anything it conjures up Thomas Mann’s *Magic Mountain* or *Death in Venice*, stations for the terminally ill rather than sites of excitement. The prevailing mood in *Grand Hotel Europe* is of decay rather than decadence, seeing entropy, a terminal winding down, in the bustle of tourism and travel. As the few remaining resident guests at the Grand Hotel Europe look at it, travel and tourism, in their present-day iteration, are forces of destruction.

When Clio and the novel’s protagonist were still together, during a visit to the Castello Mackenzie in Genoa, a nineteenth-century fantasy structure erected in mock Florentine renaissance style, their conversation touches on the theme of authenticity and its replica versions. Castello Mackenzie, fake when it was built, a nostalgic dream come true, had since been put on the list of protected architectural monuments. As Clio puts it: “This is nostalgia squared, a quadratic version of it.” Both agree that it will never be the real thing, never be truly old. The writer then adds “I think it is our European blood. This way of thinking typifies us Westerners. It is the curse of the old continent. … You could summarize the history of Europe as a history of the continued longing for history.” If the remembrance of things past constitutes the dreams we dream today, nostalgia will be the defining element of our outlook on life. And as for the quest for authenticity, taking the Mona Lisa as an example, the writer has this to say: “What matters is that people want to see the Mona Lisa *not* for the sake of the experience of seeing her in reality. What matters is what Walter Benjamin called the aura of the work of art. Or rather, not so much the work of art itself, but the sensation of being up close to it, preferably stamped with the seal of authenticity of a photograph or a selfie.” (63, 113).

We’ll leave Pfeijffer on this ironically deconstructionist view of present-day tourism. He is keenly aware of Europe as a stage for global tourism, caught in an existential battle between the urge to preserve an authentic cultural heritage, while seeing it buckle under when confronted with the many in their quest for the real thing. He also repeats a point made by others before him, by observers such as Umberto Eco or Jean Baudrillard, that often the fake is to be preferred to the real thing, the simulacrum to the authentic version. Europe’s crumbling cultural heritage is to be admired many times over in the U.S., in Las Vegas and other such places. And what is more, Europe has jumped on the bandwagon, repackaging itself in Disney-like tourist versions, for bus loads of Chinese tourists to behold and capture on cellphones. There is a hilarious TV documentary on this, made over a decade ago: Theme Park Holland (*Pretpark Nederland*).^8^

Here too the hungry beast of commercial mass tourism has discovered the value and attraction of nostalgia. It has moved up-market to cater for the tastes of tourist snobs (who will never admit to this). It now offers upscale nostalgic tours while packaging and selling the authentic historical experience. High-brow newspapers now offer city tours, 25 days, all-in, with expert guides, and the odd afternoon lecture on board the cruise ship, to places ranging from San Francisco and New York, to Athens and Berlin. They are on tantalizing display in the ads, ready for consumption. Been there, done that. The true spirit of history, the historical experience, is nowhere to be had. “Just follow the guide, please.” To visitors and locals alike, the effect is the same. To residents the place is no longer theirs, to visitors, authenticity is nowhere to be found. As Tony Perrottet, author of *Pagan Holiday*, who lives in Manhattan, put it: “God, there’s nothing more annoying than getting stuck on Fifth Avenue between a bunch of tourists.”^9^ Yet, according to him, anti-tourist sentiment can be traced at least as far back as the first and second centuries A.D., when wealthy Romans visited Greece (where they complained about the food), Naples (where they complained about the guides), and Egypt (where they defaced the pyramids and the Sphinx with graffiti). “The structure of tourism historically is that you have resentful locals, and rich, obnoxious, clueless intruders: the Greeks and the Romans, the Brits and the Americans, the Dutch and Germans.”^10^ This may suggest there being a deep structure to the trials and tribulations visited equally upon tourists and their locales over the ages. If so, it is a far cry from a more reflective mental attitude, characteristic of the traveler more than the tourist. It may put us in mind of Claude Lévi-Strauss’s ruminations in his *Tristes Tropiques* (first published in 1955). Reminiscing on his peregrinations as a researcher and traveler, he describes himself as “an archeologist of space, seeking in vain to recreate a lost local colour with the help of fragments and debris.” It inspires the following lament: “I wished I had lived in the days of *real* journeys, when it was still possible to see the full splendour of a spectacle that had not yet been blighted, polluted and spoilt.” In other words, a spectacle that had managed to stay pristine, untouched by the march of time and the ruins of tourism. Yet, as Lévi-Strauss then acknowledges, this view of things creates a false binary, suggestive of a before and after, falsely imposing a view of history as a matter of static formations succeeding each other rather than as an ongoing transformative process. “While I complain of being able to glimpse no more than the shadow of the past, I may be insensitive to reality as it is taking shape at this very moment.… A few hundred years hence … another traveller, as despairing as myself will mourn the disappearance of what I might have seen, but failed to see.”^11^

In this vein, moving now to the second part of my argument, the challenge before us will be to avoid the trap as sketched here by Lévi-Strauss and to take a fresh look at tourism as it changes shape before our eyes.

## The Pleasures of Post-Modernity

“Historical marker ahead.” Driving along America’s highways and by-ways we pass many such signs. I for one always duly stop, curious to find out what local boosters have deemed worthy of a fleeting moment’s reflection by transient travelers. That is all as it should be. Hardly ever has a historical marker served to create a true “lieu de mémoire.” Places thus marked sink back into oblivion the moment we step on the gas again. Yet, at times, the marker may become more than the impassive purveyor of tidbits of knowledge and speak to us in the active voice of a participant in history. Or shall we say: a silent witness.

A telling case is that of Emmett Till, a fourteen-year old black Chicago youngster, who when visiting his uncle in the Mississippi Delta, in 1955, was viciously murdered by whites in retaliation for allegedly having wolf-whistled at a white female shop assistant. It became one of the moments that helped to launch the Civil Rights movements in the late 1950s. Certainly a moment worthy of a historical marker. Yet rather than becoming a token of remembrance, bringing reconciliation, it drew the ire of local white supremacists, who vented their anger on the monument. In its present iteration it is the fourth marker on the spot where Emmett Till’s mutilated body was drawn from the waters. Previous markers were stolen, thrown in the river, replaced only to be riddled with bullet holes, cut down, replaced again, shot up again. The new memorial weighs 500 pounds and is made of reinforced steel covered in bullet-proof glass. It is surrounded by security cameras. Two weeks after having been put in its place six white men and two women gathered there. One carried the flag of a group called the League of the South, which advocates for “Anglo-Celtic” supremacy. Its founder, Michael Hill, said: “We are at the Emmett Till monument that represents the civil-rights movement for blacks. What we want to know is: when are all of the white people over the last fifty years that have been murdered, assaulted and raped by blacks going to be memorialized?” When the security cameras picked up the protest and triggered an alarm, the protesters ran away.

Just sixteen miles south lies Glendora, a small town that houses the largest collection of memorials, including an Emmett Till museum. Glendora is one of the poorest towns in the impoverished Mississippi Delta. There is even an NGO devoted to combating poverty in – mind you – Haiti, Guatemala, Peru – *and* Glendora. In 2009 the Mississippi Development Authority sent a team of economists to the town. After describing it as a place with “no hope,” they said its only viable asset was civil-rights tourism. In an utter twist of irony, the hope now is to bring tourists to a place meant to commemorate the plight of blacks and their civil rights struggles and to pay their respects to the historic victims of racism, when in fact the place more easily rallies its evil perpetrators.

All in all, this is not the sort of story that your average roadside historical marker has in store. If a marker put up in Glendora would tell a story, it would be a never-ending story, with no clear end in sight, no upward slope, no light at the end. If it conjures up a past nostalgically remembered, it is the wrong past of racism and white supremacy. If there is anything postmodern about this, it must be in its mass-media iterations, with a current president as ringleader who sees “good people on both sides,” and to whom there is an equivalence on all sides of moral issues. There may in fact, under America’s present political leadership, be a vast moral erosion at work, leaving everything morally polyvalent, equally plausible, equally capable of being turned into mass entertainment.

As for tourism and the urge to travel and trade places, a similar erosion may be at work. Like moral issues in the public realm tourist destinations have likewise become interchangeable, open to the total make-over that the masters of mass manipulation can give them. Exclusivity is simply a matter of claiming it in the face of mass-produced uniformity, as Don de Lillo describes his students entering to attend class: “They came in out of the sun in their limited-edition T-shirts.” Limited edition, yet mass-produced. Hypes can be created, tourist flows can be got going, directed and changed. A classic illustration of this happening is Don de Lillo’s hilarious spoof about a tourist attraction known as “the most photographed barn in America.” As he tells the story in his deadpan way, he takes a young colleague who has joined his department of Hitler Studies in Blacksmith, a god-forsaken little college town somewhere in the mountain West. “We drove twenty-two miles into the country around Farmington. There were meadows and apple orchards. White fences trailed through the rolling fields. Soon the signs started appearing. THE MOST PHOTOGRAPHED BARN IN AMERICA. We counted five signs before we reached the site. There were forty cars and a tour bus in the makeshift lot. We walked along a cowpath to the slightly elevated spot set aside for viewing and photographing. All the people had cameras; some had tripods, telephoto lenses, filter kits. A man in a booth sold postcards and slides – pictures of the barn taken from the elevated spot. We stood near a grove of trees and watched the photographers. Murray - the narrator’s young colleague – maintained a prolonged silence, occasionally scrawling some notes in a little book. “No one see the barn,” he said finally. A long silence followed. “Once you’ve seen the signs about the barn, it becomes impossible to see the barn.” He fell silent once more. People with cameras left the elevated site, replaced at once by others. “We’re not here to capture an image, we’re here to maintain one. Every photograph reinforces the aura. Can you feel it, Jack? An accumulation of nameless energies.” There was an extended silence. The man in the booth sold postcards and slides. ‘Being here is a kind of spiritual surrender. We only see what the others see. The thousands that were here in the past, those who will come in the future. We’ve agreed to be part of a collective perception. This literally colors our vision. A religious experience in a way, like all tourism. Another silence ensued. “They are taking pictures of taking pictures,” he said. He did not speak for a while. We listened to the incessant clicking of shutter release buttons, the rustling crank of levers that advanced the film. “What was the barn like before it was photographed?” he said. “What did it look like, how was it different from other barns, how was it similar to other barns? We can’t answer these questions because we’ve read the signs, seen the people snapping the pictures. We can’t get outside the aura. We’re part of the aura. We’re here, we’re now.”

He seemed immensely pleased by this.”^12^

This is probably as good an evocation as any of the pleasures of post-modernism. It is a matter of a second-order pleasure, derived from going with the flow, from doing as others do, from waiting in line to take the same picture as others just did, yet at the same time reaching transcendence, seeing yourself in the crowd, yet as if from a distance. There is a double vision involved, and a dose of irony thrown in for free. This takes us one step further than De Lillo: We are part of the aura, yet we can also at the same time step outside it. And photography is there to prove it. A beautiful illustration of this is offered by a classic photograph taken by Lee Friedlander.^13^ The tourist scene it shows is Mount Rushmore, photographed from many angles, in many reflections, blurring inside and outside. These are people who, as De Lillo would have it, are all part of the aura, all here, all now. Yet Friedlander’s photograph has become a critical ingredient in our enjoyment of the moment. He deconstructs reality for us, decomposing it into mirrorlike reflections of reflections; as in an early Cubist phantasy, he produces an ironic comment and gives us pleasure. Friedlander adds the layer of photography to the mirrorlike layers that he had chosen to photograph; the entertainment he offers is not unlike the awe we feel when confronted with the circus act of a man keeping cups and saucers up in the air. Such are the joys and pleasures offered by the asinine forms of contemporary mass tourism, if we allow ourselves to transcend it, and becoming its ironic observers. All we need to do is develop an eye and an ear for it. If Kilroy can do it, why can’t we?

Well, for one thing, as the first half of my story may remind us, the commodification paradox will keep us from over-indulging in the pleasures of post-modernity. It may serve as a sobering reality check. Admittedly, the pleasures of post-modern tourism, as here described, all have an undeniable high-brow touch, a whiff of elitism, about them. Particularly in their armchair variety where the joy lies in versions of inner, imaginary, travel, as almost a form of escapism, a form of inner emigration. At any rate, it will always be a joy for an elite and will never be able to rival the immediate exhilaration and excitement of contemporary mass tourism, from snow mobiles racing through Yellowstone Park or masses of down-hill skiers laying waste to fragile mountain meadows. Over-tourism as a force devastating every object of mass-longing is here to stay, it seems, offering no escape.

Whatever glimmers of hope there are may be too late. Movements of resistance may be gestating, centering on issues of ecological sustainability, rallying support around shared feelings of shame and guilt. Buzzwords are spreading like wildfire over the Internet, words like flight shame, originating in Sweden, are exerting downward pressure on air travel, inspiring people to reduce their carbon footprint. Even in consumer hotbeds like China, climate consciousness is on the rise. It enjoys an Instagram-fueled tailwind from successful campaigns against plastics. A global movement is gaining momentum that grants legal personhood to rivers, lakes, forests and mountains. In its American iteration the movement takes a leaf, ironically, from the long-standing corporate practice that turns corporate entities into legal persons, giving them a voice and having them speak on behalf of corporate interests. This time environmentalism is in command, speaking on behalf of threatened eco-systems, such as lakes and valleys. A recent case is the Lake Erie Ecosystem Bill of Rights, adopted by 61% of the voters in a February, 2019, referendum, granting the Lake Erie ecosystem legal personhood, with all consequent rights in law, including the right “to exist, flourish, and naturally evolve.” It joined other more-than-human entities accorded legal personhood, in India, New Zealand, the Colombian Amazon and Ecuador. All such recent legal moves have come to be known as the “natural rights” or “rights of nature” movement.^14^

Whether such movements, and the changes in the ways people think and behave about the environment, are enough to fend off the worst-case environmental scenarios, we cannot tell. Doomsday scenarios may still appear more likely outcomes, with tourism no more than an echo from a time before the ultimate cataclysm. Again, De Lillo may help us to conjure this up in our minds. Having given us a taste of the post-modern pleasures of travel in his passages about the most-photographed barn in its quasi-Arcadian setting, the setting changes to one where cataclysm has struck. We enter familiar De Lillo terrain, as in his *Cosmopolis*, or *Falling Man.* In an inspired moment the story line turns back on itself - with the central characters fleeing from the toxic cloud that hangs over Blacksmith, leaking from a derailed tank car in the railroad yard - they pass a road sign, pointing them to the most photographed barn in America. Tourism has overnight turned into a flight for one’s life, in a nightmare world reminiscent of literary evocations like Cormac McCarthy’s *The Road* or Jonathan Raban’s *Surveillance.*^15^ No more postmodern pleasures to be had here, nor are any on offer.

## Footnotes

1 Henry James, The American Scene, quoted from etext of *The American Scene* (London, Chapman & Hall, ltd., 1907), at http://www2.newpaltz.edu/~hathawar/americanscene2.html. Last accessed January 4th, 2020, pp. 84,85.

2 Rene Chun, “The Dutch War on Tourists,” https://www.theatlantic.com/magazine/archive/2019/09/the-war-on-tourists/594766/. Last accessed January 4th, 2020.

3 Lawrence N. Powell, *New Orleans: An American Pompeii?* In: Reinhold Wagnleitner, ed., *Satchmo Meets Amadeus* (Innsbruck: Studienverlag, 2006) 147.

4 John Taliaferro, *Grinnell: America’s Environmental Pioneer and His Restless Drive to Save the West.* (New York: Liveright/Norton, 2019).

5 Bill Bryson, *A Walk in the Woods*: *Rediscovering America on the Appalachian Trail* (New York: Broadway Books, 1998); Jonathan Raban, *Hunting Mr Heartbreak* (London: Collins Harvill, 1990).

6 Stefan Zweig, *The World of Yesterday* (translated from German by Anthea Bell) (Lincoln: University of Nebraska Press, 2013).

7 Ilja Leonard Pfeijffer, *Grand-Hotel Europa* (Amsterdam: Arbeiderspers, 2019) References – in brackets – are to pages in the Dutch version. Translations are mine.

8 Michiel van Erp, *Pretpark Nederland* (TV documentary, VPRO Television, 2006).

9 Tony Perrottet, *Pagan Holiday: On the Trail of Ancient Roman Tourists* (New York: Random House, 2003).

10 Perrottet, as quoted in Chun, *The Atlantic* (cf. footnote 2, above).

11 Claude Lévi-Strauss, *Tristes Tropiques* (1955) London: Penguin Books, 1992 (translation by John Weightman, and Doreen Weightman; Introd. and Notes by Patrick Wilckens) pp. 43, 44, 45.

12 Don DeLillo, *White Noise* (1984) London: Picador, 2011, quotations from pp. 13-15, 30.

13 Go to https://sites.middlebury.edu/landandlens/2016/10/16/lee-friedlander-mt-rushmore-south-dakota-1969/

14 Robert MacFarlane, “Should this valley have rights?”, *The Guardian Weekly,* 8 November 2019 (https://www.theguardian.com/books/2019/nov/02/trees-have-rights-too-robert-macfarlane-on-the-new-laws-of-nature) Also: Stone, Christopher D., “Should Trees Have Standing? Towards Legal Rights for Natural Objects,” *Southern California Law Review*, 45 (1972): 450-501 For a discussion of the rise of corporate legal persons and their disembodied voices, see my “The Revenge of the Simulacrum: The Reality Principle Meets Reality TV,” *Social Science and Modern Society*, 56, 5, (September/October 2019): 419-427.

15 Cormac McCarthy, *The Road* (New York: Random House, 2007), Jonathan Raban, *Surveillance* (New York: Pantheon Books, 2006).

